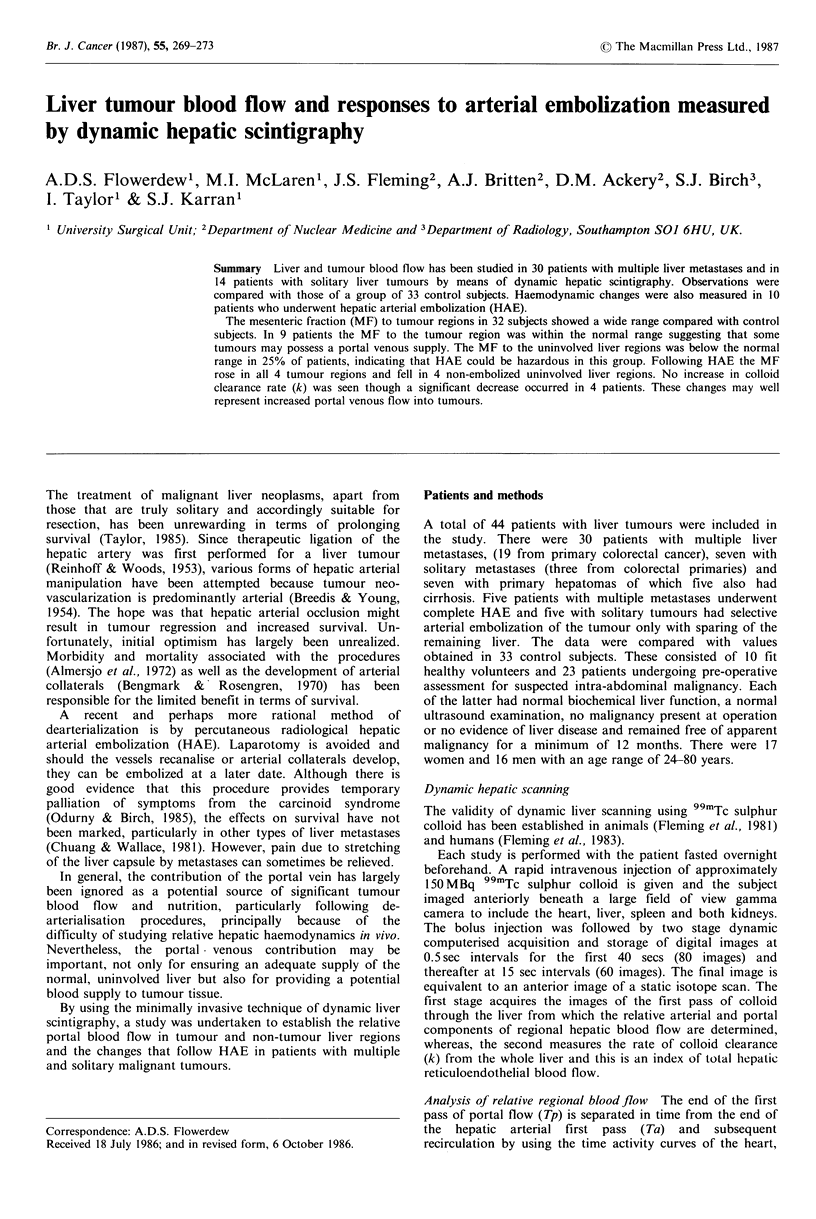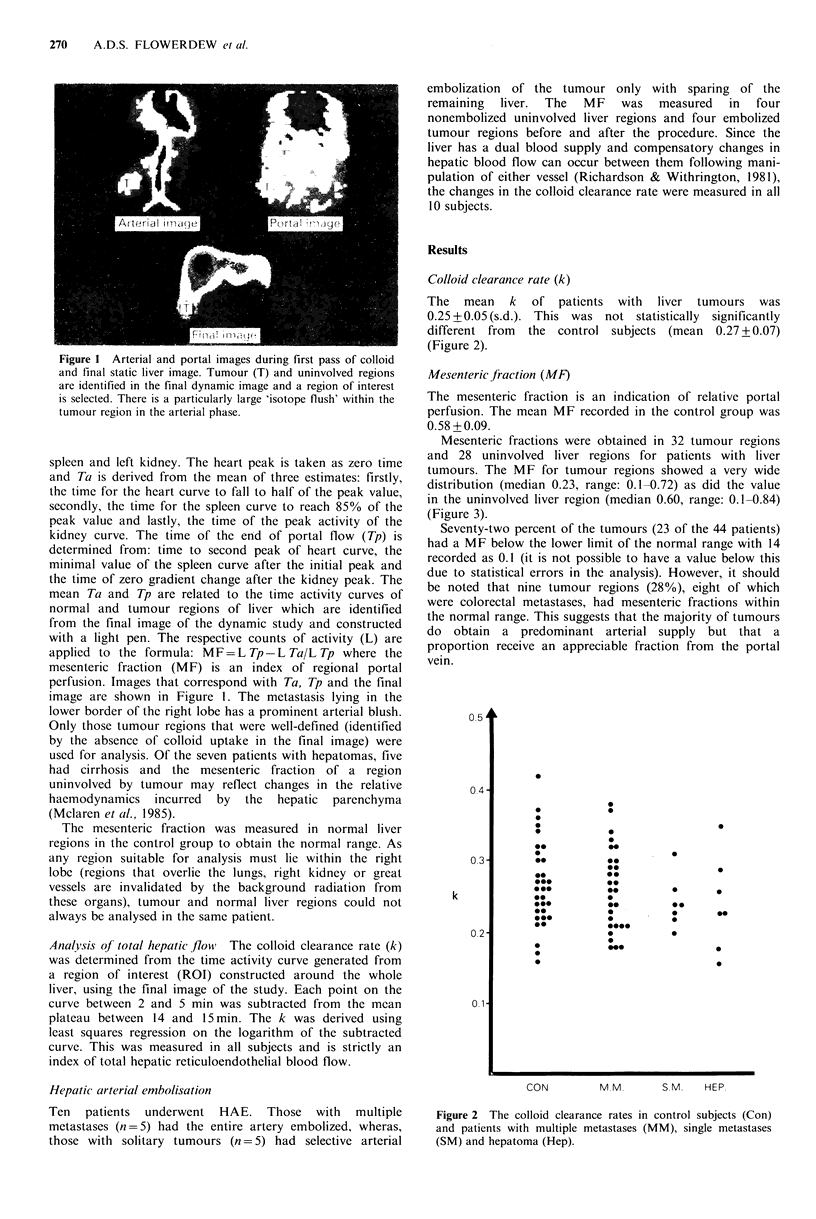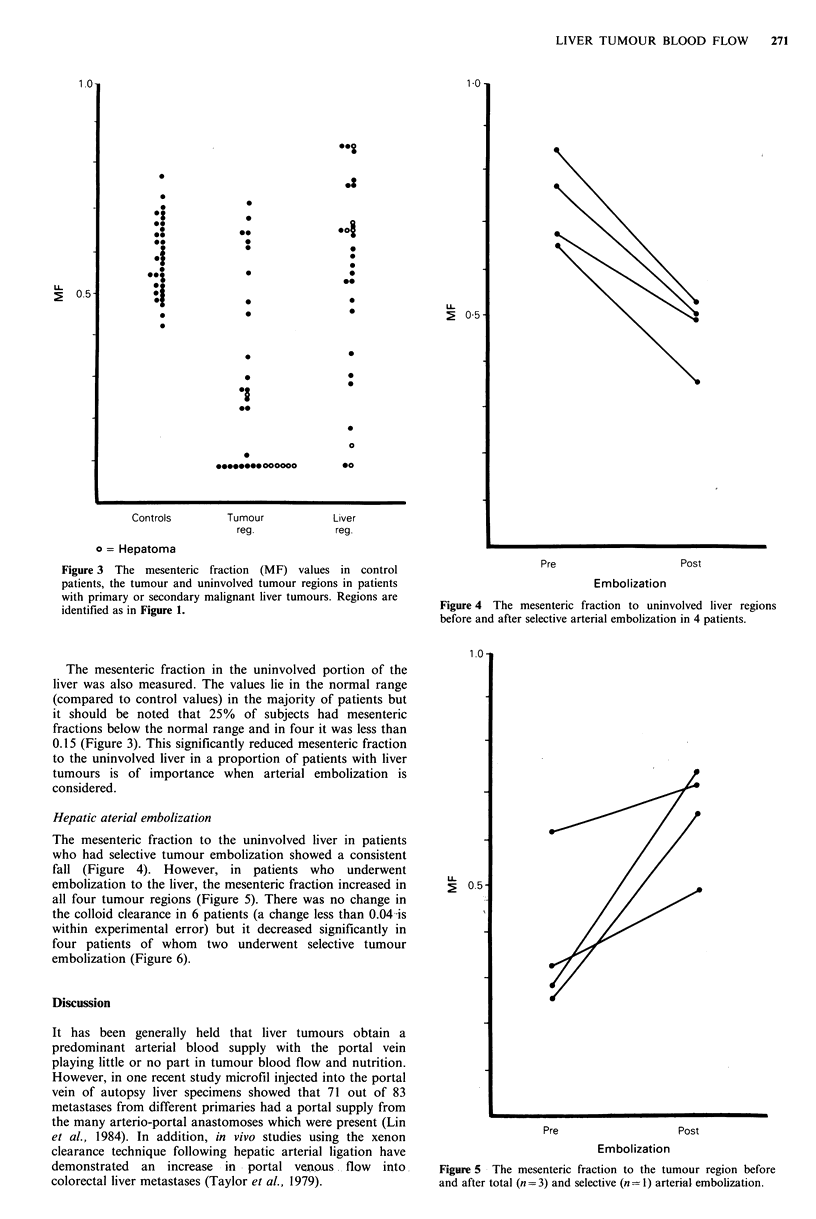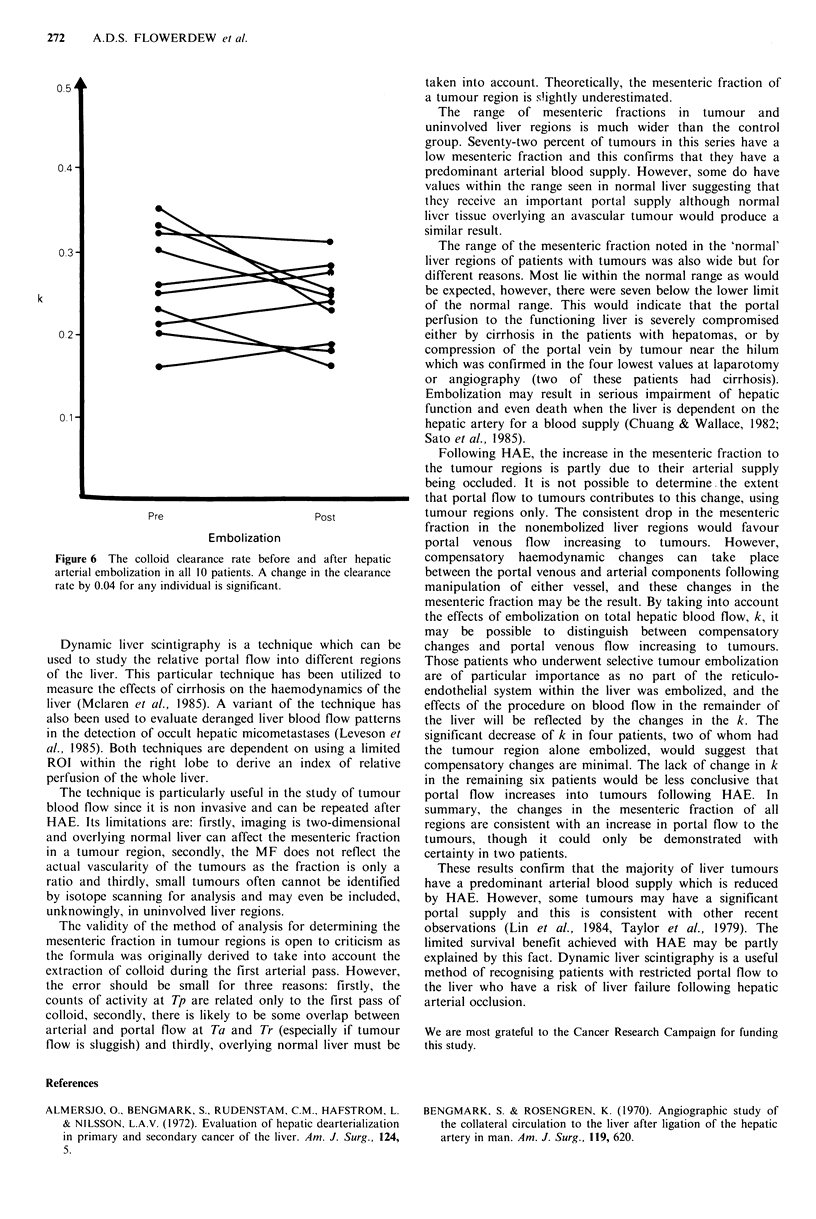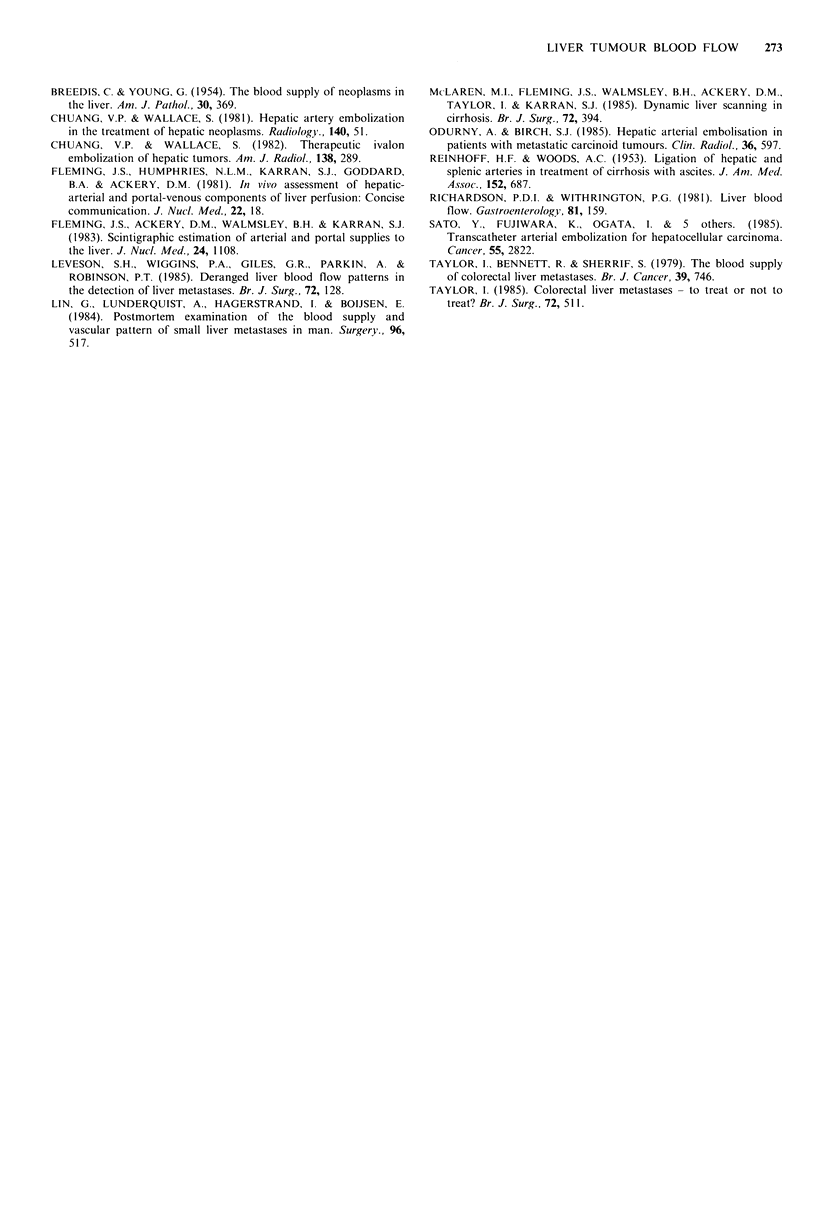# Liver tumour blood flow and responses to arterial embolization measured by dynamic hepatic scintigraphy.

**DOI:** 10.1038/bjc.1987.52

**Published:** 1987-03

**Authors:** A. D. Flowerdew, M. I. McLaren, J. S. Fleming, A. J. Britten, D. M. Ackery, S. J. Birch, I. Taylor, S. J. Karran

## Abstract

**Images:**


Br. J. Cancer (1987), 55, 269–273
© The Macmillan Press Ltd., 1987

### Liver tumour blood flow and responses to arterial embolization measured by dynamic hepatic scintigraphy

A.D.S. Flowerdew[1], M.I. McLaren[1], J.S. Fleming[2], A.J. Britten[2], D.M. Ackery[2], S.J. Birch[3], I. Taylor[1] & S.J. Karran[1]

[1] *University Surgical Unit;* [2]*Department of Nuclear Medicine and* [3]*Department of Radiology, Southampton SO1 6HU, UK.*

**Summary** Liver and tumour blood flow has been studied in 30 patients with multiple liver metastases and in 14 patients with solitary liver tumours by means of dynamic hepatic scintigraphy. Observations were compared with those of a group of 33 control subjects. Haemodynamic changes were also measured in 10 patients who underwent hepatic arterial embolization (HAE).

The mesenteric fraction (MF) to tumour regions in 32 subjects showed a wide range compared with control subjects. In 9 patients the MF to the tumour region was within the normal range suggesting that some tumours may possess a portal venous supply. The MF to the uninvolved liver regions was below the normal range in 25% of patients, indicating that HAE could be hazardous in this group. Following HAE the MF rose in all 4 tumour regions and fell in 4 non-embolized uninvolved liver regions. No increase in colloid clearance rate ($k$) was seen though a significant decrease occurred in 4 patients. These changes may well represent increased portal venous flow into tumours.

The treatment of malignant liver neoplasms, apart from those that are truly solitary and accordingly suitable for resection, has been unrewarding in terms of prolonging survival (Taylor, 1985). Since therapeutic ligation of the hepatic artery was first performed for a liver tumour (Reinhoff & Woods, 1953), various forms of hepatic arterial manipulation have been attempted because tumour neovascularization is predominantly arterial (Breedis & Young, 1954). The hope was that hepatic arterial occlusion might result in tumour regression and increased survival. Unfortunately, initial optimism has largely been unrealized. Morbidity and mortality associated with the procedures (Almersjo *et al.*, 1972) as well as the development of arterial collaterals (Bengmark & Rosengren, 1970) has been responsible for the limited benefit in terms of survival.

A recent and perhaps more rational method of dearterialization is by percutaneous radiological hepatic arterial embolization (HAE). Laparotomy is avoided and should the vessels recanalise or arterial collaterals develop, they can be embolized at a later date. Although there is good evidence that this procedure provides temporary palliation of symptoms from the carcinoid syndrome (Odurny & Birch, 1985), the effects on survival have not been marked, particularly in other types of liver metastases (Chuang & Wallace, 1981). However, pain due to stretching of the liver capsule by metastases can sometimes be relieved.

In general, the contribution of the portal vein has largely been ignored as a potential source of significant tumour blood flow and nutrition, particularly following dearterialisation procedures, principally because of the difficulty of studying relative hepatic haemodynamics *in vivo*. Nevertheless, the portal venous contribution may be important, not only for ensuring an adequate supply of the normal, uninvolved liver but also for providing a potential blood supply to tumour tissue.

By using the minimally invasive technique of dynamic liver scintigraphy, a study was undertaken to establish the relative portal blood flow in tumour and non-tumour liver regions and the changes that follow HAE in patients with multiple and solitary malignant tumours.

Correspondence: A.D.S. Flowerdew
Received 18 July 1986; and in revised form, 6 October 1986.

### Patients and methods

A total of 44 patients with liver tumours were included in the study. There were 30 patients with multiple liver metastases, (19 from primary colorectal cancer), seven with solitary metastases (three from colorectal primaries) and seven with primary hepatomas of which five also had cirrhosis. Five patients with multiple metastases underwent complete HAE and five with solitary tumours had selective arterial embolization of the tumour only with sparing of the remaining liver. The data were compared with values obtained in 33 control subjects. These consisted of 10 fit healthy volunteers and 23 patients undergoing pre-operative assessment for suspected intra-abdominal malignancy. Each of the latter had normal biochemical liver function, a normal ultrasound examination, no malignancy present at operation or no evidence of liver disease and remained free of apparent malignancy for a minimum of 12 months. There were 17 women and 16 men with an age range of 24–80 years.

### *Dynamic hepatic scanning*

The validity of dynamic liver scanning using $^{99m}$Tc sulphur colloid has been established in animals (Fleming *et al.*, 1981) and humans (Fleming *et al.*, 1983).

Each study is performed with the patient fasted overnight beforehand. A rapid intravenous injection of approximately 150 MBq $^{99m}$Tc sulphur colloid is given and the subject imaged anteriorly beneath a large field of view gamma camera to include the heart, liver, spleen and both kidneys. The bolus injection was followed by two stage dynamic computerised acquisition and storage of digital images at 0.5 sec intervals for the first 40 secs (80 images) and thereafter at 15 sec intervals (60 images). The final image is equivalent to an anterior image of a static isotope scan. The first stage acquires the images of the first pass of colloid through the liver from which the relative arterial and portal components of regional hepatic blood flow are determined, whereas, the second measures the rate of colloid clearance ($k$) from the whole liver and this is an index of total hepatic reticuloendothelial blood flow.

*Analysis of relative regional blood flow* The end of the first pass of portal flow ($Tp$) is separated in time from the end of the hepatic arterial first pass ($Ta$) and subsequent recirculation by using the time activity curves of the heart,

**Figure 1** Arterial and portal images during first pass of colloid and final static liver image. Tumour (T) and uninvolved regions are identified in the final dynamic image and a region of interest is selected. There is a particularly large 'isotope flush' within the tumour region in the arterial phase.

spleen and left kidney. The heart peak is taken as zero time and *Ta* is derived from the mean of three estimates: firstly, the time for the heart curve to fall to half of the peak value, secondly, the time for the spleen curve to reach 85% of the peak value and lastly, the time of the peak activity of the kidney curve. The time of the end of portal flow (*Tp*) is determined from: time to second peak of heart curve, the minimal value of the spleen curve after the initial peak and the time of zero gradient change after the kidney peak. The mean *Ta* and *Tp* are related to the time activity curves of normal and tumour regions of liver which are identified from the final image of the dynamic study and constructed with a light pen. The respective counts of activity (L) are applied to the formula: $MF = L\,Tp - L\,Ta / L\,Tp$ where the mesenteric fraction (MF) is an index of regional portal perfusion. Images that correspond with *Ta, Tp* and the final image are shown in Figure 1. The metastasis lying in the lower border of the right lobe has a prominent arterial blush. Only those tumour regions that were well-defined (identified by the absence of colloid uptake in the final image) were used for analysis. Of the seven patients with hepatomas, five had cirrhosis and the mesenteric fraction of a region uninvolved by tumour may reflect changes in the relative haemodynamics incurred by the hepatic parenchyma (Mclaren *et al.*, 1985).

The mesenteric fraction was measured in normal liver regions in the control group to obtain the normal range. As any region suitable for analysis must lie within the right lobe (regions that overlie the lungs, right kidney or great vessels are invalidated by the background radiation from these organs), tumour and normal liver regions could not always be analysed in the same patient.

*Analysis of total hepatic flow* The colloid clearance rate (*k*) was determined from the time activity curve generated from a region of interest (ROI) constructed around the whole liver, using the final image of the study. Each point on the curve between 2 and 5 min was subtracted from the mean plateau between 14 and 15 min. The *k* was derived using least squares regression on the logarithm of the subtracted curve. This was measured in all subjects and is strictly an index of total hepatic reticuloendothelial blood flow.

*Hepatic arterial embolisation*

Ten patients underwent HAE. Those with multiple metastases ($n = 5$) had the entire artery embolized, wheras, those with solitary tumours ($n = 5$) had selective arterial embolization of the tumour only with sparing of the remaining liver. The MF was measured in four nonembolized uninvolved liver regions and four embolized tumour regions before and after the procedure. Since the liver has a dual blood supply and compensatory changes in hepatic blood flow can occur between them following manipulation of either vessel (Richardson & Withrington, 1981), the changes in the colloid clearance rate were measured in all 10 subjects.

### Results

*Colloid clearance rate* (*k*)

The mean *k* of patients with liver tumours was $0.25 \pm 0.05$ (s.d.). This was not statistically significantly different from the control subjects (mean $0.27 \pm 0.07$) (Figure 2).

*Mesenteric fraction* (*MF*)

The mesenteric fraction is an indication of relative portal perfusion. The mean MF recorded in the control group was $0.58 \pm 0.09$.

Mesenteric fractions were obtained in 32 tumour regions and 28 uninvolved liver regions for patients with liver tumours. The MF for tumour regions showed a very wide distribution (median 0.23, range: 0.1–0.72) as did the value in the uninvolved liver region (median 0.60, range: 0.1–0.84) (Figure 3).

Seventy-two percent of the tumours (23 of the 44 patients) had a MF below the lower limit of the normal range with 14 recorded as 0.1 (it is not possible to have a value below this due to statistical errors in the analysis). However, it should be noted that nine tumour regions (28%), eight of which were colorectal metastases, had mesenteric fractions within the normal range. This suggests that the majority of tumours do obtain a predominant arterial supply but that a proportion receive an appreciable fraction from the portal vein.

**Figure 2** The colloid clearance rates in control subjects (Con) and patients with multiple metastases (MM), single metastases (SM) and hepatoma (Hep).

**Figure 3** The mesenteric fraction (MF) values in control patients, the tumour and uninvolved tumour regions in patients with primary or secondary malignant liver tumours. Regions are identified as in **Figure 1.**

**Figure 4** The mesenteric fraction to uninvolved liver regions before and after selective arterial embolization in 4 patients.

The mesenteric fraction in the uninvolved portion of the liver was also measured. The values lie in the normal range (compared to control values) in the majority of patients but it should be noted that 25% of subjects had mesenteric fractions below the normal range and in four it was less than 0.15 (Figure 3). This significantly reduced mesenteric fraction to the uninvolved liver in a proportion of patients with liver tumours is of importance when arterial embolization is considered.

*Hepatic aterial embolization*

The mesenteric fraction to the uninvolved liver in patients who had selective tumour embolization showed a consistent fall (Figure 4). However, in patients who underwent embolization to the liver, the mesenteric fraction increased in all four tumour regions (Figure 5). There was no change in the colloid clearance in 6 patients (a change less than 0.04 is within experimental error) but it decreased significantly in four patients of whom two underwent selective tumour embolization (Figure 6).

### Discussion

It has been generally held that liver tumours obtain a predominant arterial blood supply with the portal vein playing little or no part in tumour blood flow and nutrition. However, in one recent study microfil injected into the portal vein of autopsy liver specimens showed that 71 out of 83 metastases from different primaries had a portal supply from the many arterio-portal anastomoses which were present (Lin *et al.*, 1984). In addition, *in vivo* studies using the xenon clearance technique following hepatic arterial ligation have demonstrated an increase in portal venous flow into colorectal liver metastases (Taylor *et al.*, 1979).

**Figure 5** The mesenteric fraction to the tumour region before and after total ($n=3$) and selective ($n=1$) arterial embolization.

**Figure 6** The colloid clearance rate before and after hepatic arterial embolization in all 10 patients. A change in the clearance rate by 0.04 for any individual is significant.

Dynamic liver scintigraphy is a technique which can be used to study the relative portal flow into different regions of the liver. This particular technique has been utilized to measure the effects of cirrhosis on the haemodynamics of the liver (Mclaren *et al.*, 1985). A variant of the technique has also been used to evaluate deranged liver blood flow patterns in the detection of occult hepatic micometastases (Leveson *et al.*, 1985). Both techniques are dependent on using a limited ROI within the right lobe to derive an index of relative perfusion of the whole liver.

The technique is particularly useful in the study of tumour blood flow since it is non invasive and can be repeated after HAE. Its limitations are: firstly, imaging is two-dimensional and overlying normal liver can affect the mesenteric fraction in a tumour region, secondly, the MF does not reflect the actual vascularity of the tumours as the fraction is only a ratio and thirdly, small tumours often cannot be identified by isotope scanning for analysis and may even be included, unknowingly, in uninvolved liver regions.

The validity of the method of analysis for determining the mesenteric fraction in tumour regions is open to criticism as the formula was originally derived to take into account the extraction of colloid during the first arterial pass. However, the error should be small for three reasons: firstly, the counts of activity at *Tp* are related only to the first pass of colloid, secondly, there is likely to be some overlap between arterial and portal flow at *Ta* and *Tr* (especially if tumour flow is sluggish) and thirdly, overlying normal liver must be taken into account. Theoretically, the mesenteric fraction of a tumour region is slightly underestimated.

The range of mesenteric fractions in tumour and uninvolved liver regions is much wider than the control group. Seventy-two percent of tumours in this series have a low mesenteric fraction and this confirms that they have a predominant arterial blood supply. However, some do have values within the range seen in normal liver suggesting that they receive an important portal supply although normal liver tissue overlying an avascular tumour would produce a similar result.

The range of the mesenteric fraction noted in the 'normal' liver regions of patients with tumours was also wide but for different reasons. Most lie within the normal range as would be expected, however, there were seven below the lower limit of the normal range. This would indicate that the portal perfusion to the functioning liver is severely compromised either by cirrhosis in the patients with hepatomas, or by compression of the portal vein by tumour near the hilum which was confirmed in the four lowest values at laparotomy or angiography (two of these patients had cirrhosis). Embolization may result in serious impairment of hepatic function and even death when the liver is dependent on the hepatic artery for a blood supply (Chuang & Wallace, 1982; Sato *et al.*, 1985).

Following HAE, the increase in the mesenteric fraction to the tumour regions is partly due to their arterial supply being occluded. It is not possible to determine the extent that portal flow to tumours contributes to this change, using tumour regions only. The consistent drop in the mesenteric fraction in the nonembolized liver regions would favour portal venous flow increasing to tumours. However, compensatory haemodynamic changes can take place between the portal venous and arterial components following manipulation of either vessel, and these changes in the mesenteric fraction may be the result. By taking into account the effects of embolization on total hepatic blood flow, *k*, it may be possible to distinguish between compensatory changes and portal venous flow increasing to tumours. Those patients who underwent selective tumour embolization are of particular importance as no part of the reticulo-endothelial system within the liver was embolized, and the effects of the procedure on blood flow in the remainder of the liver will be reflected by the changes in the *k*. The significant decrease of *k* in four patients, two of whom had the tumour region alone embolized, would suggest that compensatory changes are minimal. The lack of change in *k* in the remaining six patients would be less conclusive that portal flow increases into tumours following HAE. In summary, the changes in the mesenteric fraction of all regions are consistent with an increase in portal flow to the tumours, though it could only be demonstrated with certainty in two patients.

These results confirm that the majority of liver tumours have a predominant arterial blood supply which is reduced by HAE. However, some tumours may have a significant portal supply and this is consistent with other recent observations (Lin *et al.*, 1984, Taylor *et al.*, 1979). The limited survival benefit achieved with HAE may be partly explained by this fact. Dynamic liver scintigraphy is a useful method of recognising patients with restricted portal flow to the liver who have a risk of liver failure following hepatic arterial occlusion.

We are most grateful to the Cancer Research Campaign for funding this study.

### References

ALMERSJO, O., BENGMARK, S., RUDENSTAM, C.M., HAFSTROM, L. & NILSSON, L.A.V. (1972). Evaluation of hepatic dearterialization in primary and secondary cancer of the liver. *Am. J. Surg.*, **124**, 5.

BENGMARK, S. & ROSENGREN, K. (1970). Angiographic study of the collateral circulation to the liver after ligation of the hepatic artery in man. *Am. J. Surg.*, **119**, 620.

BREEDIS, C. & YOUNG, G. (1954). The blood supply of neoplasms in the liver. *Am. J. Pathol.*, **30**, 369.

CHUANG, V.P. & WALLACE, S. (1981). Hepatic artery embolization in the treatment of hepatic neoplasms. *Radiology.*, **140**, 51.

CHUANG, V.P. & WALLACE, S. (1982). Therapeutic ivalon embolization of hepatic tumors. *Am. J. Radiol.*, **138**, 289.

FLEMING, J.S., HUMPHRIES, N.L.M., KARRAN, S.J., GODDARD, B.A. & ACKERY, D.M. (1981). *In vivo* assessment of hepatic-arterial and portal-venous components of liver perfusion: Concise communication. *J. Nucl. Med.*, **22**, 18.

FLEMING, J.S., ACKERY, D.M., WALMSLEY, B.H. & KARRAN, S.J. (1983). Scintigraphic estimation of arterial and portal supplies to the liver. *J. Nucl. Med.*, **24**, 1108.

LEVESON, S.H., WIGGINS, P.A., GILES, G.R., PARKIN, A. & ROBINSON, P.T. (1985). Deranged liver blood flow patterns in the detection of liver metastases. *Br. J. Surg.*, **72**, 128.

LIN, G., LUNDERQUIST, A., HAGERSTRAND, I. & BOIJSEN, E. (1984). Postmortem examination of the blood supply and vascular pattern of small liver metastases in man. *Surgery.*, **96**, 517.

McLAREN, M.I., FLEMING, J.S., WALMSLEY, B.H., ACKERY, D.M., TAYLOR, I. & KARRAN, S.J. (1985). Dynamic liver scanning in cirrhosis. *Br. J. Surg.*, **72**, 394.

ODURNY, A. & BIRCH, S.J. (1985). Hepatic arterial embolisation in patients with metastatic carcinoid tumours. *Clin. Radiol.*, **36**, 597.

REINHOFF, H.F. & WOODS, A.C. (1953). Ligation of hepatic and splenic arteries in treatment of cirrhosis with ascites. *J. Am. Med. Assoc.*, **152**, 687.

RICHARDSON, P.D.I. & WITHRINGTON, P.G. (1981). Liver blood flow. *Gastroenterology*, **81**, 159.

SATO, Y., FUJIWARA, K., OGATA, I. & 5 others. (1985). Transcatheter arterial embolization for hepatocellular carcinoma. *Cancer*, **55**, 2822.

TAYLOR, I., BENNETT, R. & SHERRIF, S. (1979). The blood supply of colorectal liver metastases. *Br. J. Cancer*, **39**, 746.

TAYLOR, I. (1985). Colorectal liver metastases – to treat or not to treat? *Br. J. Surg.*, **72**, 511.